# A novel method of BiFormer with temporal-spatial characteristics for ECG-based PVC detection

**DOI:** 10.3389/fphys.2025.1549380

**Published:** 2025-05-20

**Authors:** Siyuan Chen, Zhen Wang, Hao Wang, Shuai Wang, Yang Li, Bing Wang

**Affiliations:** ^1^ The First Clinical Medical College, Heilongjiang University of Chinese Medicine, Harbin, China; ^2^ First Affiliated Hospital, Heilongjiang University of Chinese Medicine, Harbin, China; ^3^ College of Computer Science and Technology, Harbin Engineering University, Harbin, China

**Keywords:** premature ventricular contraction, deep learning, electrocardiogram, Markov transition field, BiFormer

## Abstract

**Introduction:**

Premature Ventricular Contractions (PVCs) can be warning signs for serious cardiac conditions, and early detection is essential for preventing complications. The use of deep learning models in electrocardiogram (ECG) analysis has aided more accurate and efficient PVC identification. These models automatically extract and analyze complex signal features, providing valuable clinical decision-making support. Here, we conducted a study focused on the practical applications of is technology.

**Methods:**

We first used the MIT-BIH arrhythmia database and a sparse low-rank algorithm to denoise ECG signals. We then transformed the one-dimensional time-series signals into two-dimensional images using Markov Transition Fields (MTFs), considering state transition probabilities and spatial location information to comprehensively capture signal features. Finally, we used the BiFormer classification model, which employs a Bi-level Routing Attention (BRA) mechanism to construct region-level affinity graphs, to retain only the regions highly relevant to our query. This approach filtered out redundant information, and optimized both computational efficiency and memory usage.

**Results:**

Our algorithm achieved a detection accuracy of 99.45%, outperforming other commonly-used PVC detection algorithms.

**Discussion:**

By integrating MTF and BiFormer, we effectively detected PVCs, facilitating an increased convergence between medicine and deep learning technology. We hope our model can help contribute to more accurate computational support for PVC diagnosis and treatment.

## 1 Introduction

Arrhythmia is one of the key manifestations of cardiovascular diseases, with premature ventricular contractions (PVCs) being particularly prevalent worldwide (Wang, 2021; Wang et al., 2021). Studies have reported that the detection rate of PVCs among patients undergoing Holter monitoring can reach up to 75% (Panizo et al., 2018). They arise from ventricular ectopic beats, which alter electrical impulse conduction pathways, resulting in wide and distorted QRS complexes (Abdalla et al., 2019). Frequent and prolonged PVCs can lead to a series of severe cardiovascular complications, such as myocardial infarction, heart failure, and even sudden cardiac arrest (Xu et al., 2024). Moreover, existing research has indicated that PVCs are closely associated with cardiomyopathy (Myadam et al., 2022). Persistent ventricular premature depolarization can lead to left ventricular structural remodeling and impaired contractile function, ultimately progressing to PVC-induced cardiomyopathy (PVICM) (Shen et al., 2023). Furthermore, studies have shown that when the PVC burden exceeds 0.12%, the patient’s risk of mortality may increase by as much as 31% (Dukes et al., 2015). However, PVCs can be quite subtle, and many patients overlook or misinterpret their symptoms, which can lead to potentially serious consequences. Thus, timely and accurate detection of PVCs is crucial for diagnosing and preventing potential cardiac risks.

Electrocardiography (ECG) is cheap and non-invasive, and has become one of the most common methods for detecting cardiac disease (Jangra et al., 2021; Bachtiger et al., 2021). However, ECG data are often subject to various noise interferences, and preprocessing signals to enhance quality is highly important. The Sparse Low-Rank Filter (SLRF) (Karthikeyani et al., 2024) is an effective signal denoising tool. Its sparsity helps identify and remove irregular and isolated noise points, and then simplifies the original signal to a representation with only a few non-zero elements, which reduces the computational complexity of data processing. The low-rank aspect focuses on the global structure of the signal, revealing the data’s main features and reducing redundancy. Thus, together, SLRF’s sparsity and low-rank properties enhance signals and lead to improved denoising performance.

ECG data are one-dimensional time series, and are associated with certain limitations in classification and detection tasks (He et al., 2019; Li et al., 2022). Specifically, one-dimensional data can only capture continuous changes and cannot effectively reflect local features, leading to informational loss. Thus, we chose to encode these time series data into a higher-dimensional image format. This transformation preserved temporal information and leveraged the successful architectures of deep learning in computer vision to identify complex structures within the time series. Several studies have converted ECG signal time series into images to reflect complex features. Deng et al. (2024) utilized Continuous Wavelet Transform (CWT) for time-frequency representation to effectively detect atrial fibrillation. CWT can simultaneously analyze signals in both the time and frequency domains, providing multi-resolution analysis and capturing signal variations across different frequency ranges, thereby more accurately extracting key features from the electrocardiogram signal. Ammour et al. (2023) employed Short-Time Fourier Transform (STFT) for feature extraction. By segmenting the ECG signal into smaller windows and then applying STFT to each segment, the signal is transformed from the time domain to the frequency domain, generating a time-frequency spectrogram. Spectrum features are extracted from the time-frequency spectrogram, enabling effective description of the properties of ECG signals. Markov Transition Field (MTF) (Wang and Oates, 2015), a complex and flexible feature extraction method, was used to construct signal spatial structures, where each signal point represented both the voltage at a specific time and the transition probabilities with other signal points. These changes provided additional information about the signal’s dynamic behavior, captured changes in local waveforms, and enhanced the expression of complex features. MTF retains the temporal correlation of the original signal across different time intervals by considering the dependency between each quantile and time step. MTF also effectively models each signal’s temporal dependency, allowing it to handle a variety of time series data and making it suitable for stationary and non-stationary signals. Additionally, by calculating transition probabilities, MTF effectively suppresses irrelevant signal fluctuations and highlights significant changes within it. Compared to existing methods, MTF can precisely capture subtle pathological changes, enhancing the ability to process complex signals, reducing human bias and diagnostic errors, and improving the accuracy of disease diagnosis and treatment outcomes. This meets the clinical demand for efficient and precise diagnosis.

In clinical practice, with the continuous advancement of physiological signal acquisition technologies, the monitoring scope and duration of physiological signals such as ECGs have significantly expanded, leading to a substantial increase in data volume. These signals are typically characterized by high dimensionality, strong temporal dependencies, and non-stationarity, resulting in a marked increase in data complexity. Efficiently processing such large-scale and complex data to extract diagnostically valuable information has become a critical challenge in the field of intelligent analysis of physiological signals. In recent years, deep learning methods have demonstrated excellent performance in automated ECG analysis due to their powerful data representation and learning capabilities, and have gradually emerged as a research focus. In particular, Vision Transformer (ViT) have significant signal detection advantages, both because they have channel-wise Multi-Layer Perceptron (MLP) blocks that can be used for per-location embedding, and because they harness attention mechanisms to model relationships across different positions (Vaswani et al., 2017). Currently, many related works have effectively implemented PVC detection based on the Transformer architecture. Nakasone et al. (2024) proposed a hybrid method combining ResNet50 and Transformer models, achieving promising results in PVC detection. The convolutional layers of ResNet50 effectively extract spatial features from ECG signals, while the Transformer leverages its self-attention mechanism to further enhance the capture of temporal information. This multi-level feature fusion allows the model to classify PVC signals with greater accuracy. Meng et al. (2022) combined the LightConv Attention (LCA) structure with CNN and attention mechanisms to achieve PVC detection. By embedding CNN with attention mechanisms, the model strengthens the weighting of critical heartbeat morphology features in ECG signals, enabling effective capture of PVC-related features. One of the key attributes of Transformers are their global receptive fields, which can effectively capture long-range dependencies. However, this capability requires pairing each input position with all other positions prior to calculation, resulting in a computational complexity that grows quadratically with the scale of the input data. Consequently, the high computational complexity of Transformers has garnered considerable research attention. Specifically, an increasing number of researchers have sought to alleviate memory pressure by incorporating sparse attention into ViT (Liu et al., 2021; Wang et al., 2023; Tu et al., 2022). However, traditional sparse attention models rely on fixed sparse patterns, and cannot dynamically adjust attention allocation based on input data, which limits models’ expressive power. To address these issues, we introduced BiFormer, a state-of-the-art Transformer model which harnesses a dynamic attention approach to enhance model flexibility and computational efficiency by adapting to different inputs (Zhu et al., 2023). The BiFormer model uses a BRA mechanism as its fundamental building block. It first trims the constructed region-level affinity graph, retaining only the top k most relevant regions in each area, which efficiently filters the most pertinent key-value pairs at a coarse-grained regional level. Fine-grained token-to-token attention is then applied to the remaining candidate region set. However, these tokens are distributed across different regions, which presents significant computational challenges. Thus, to improve computational efficiency, we collected the dispersed tokens and performed dense matrix multiplication. Moreover, BiFormer aligns well with the structural characteristics and clinical diagnostic requirements of ECG signals. ECG signals not only exhibit distinct local waveform structures and periodic fluctuations, but also demonstrate long-range dependencies across cycles. BiFormer effectively extracts local key features at the regional level through a hierarchical attention mechanism, while utilizing token-level dynamic attention to precisely capture global rhythm information, which perfectly matches the local-global nature of ECG signals. On the other hand, in addressing the high non-stationarity of ECG signals, BiFormer’s dynamic sparse mechanism adapts the attention connections based on the input, allowing the model to more effectively focus on critical diagnostic bands, thereby enhancing its discriminative power and generalization performance. Therefore, BiFormer exhibits significant advantages in terms of modeling capacity, adaptability, and computational efficiency, making it particularly well-suited for ECG classification tasks and motivating its application in our study.

Here, we first applied SLRF to denoise the ECG data and enhance its quality. We next used MTF to transform the one-dimensional time series data into two-dimensional images, enabling a more comprehensive capture of the ECG’s spatiotemporal features. We then used the BiFormer model’s BRA mechanism to optimize information processing for key regions. Experimental results indicate that our proposed method outperforms previous approaches in effective PVC detection.

## 2 Materials and methods

**FIGURE 1:**
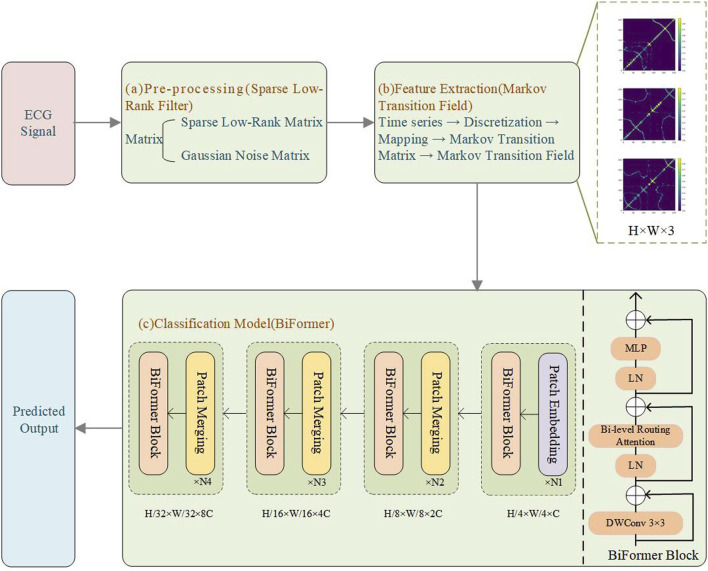
Experimental flowchart. **(a)** Pre-processing (SLRF); **(b)** Feature extraction (MTF); **(c)** Classification model (BiFormer).

Early PVC detection is crucial for preventing adverse cardiovascular outcomes, especially in high-risk populations. Early detection can help point out structural cardiac abnormalities, allowing for timely interventions and treatments that reduce the risk of more severe complications. Thus, we propose a novel PVC detection framework, illustrated in Figure 1, which consists of three components. Module (a) involves preprocessing data using SLRF to enhance ECG signal quality. We begin by collecting input signals from the MIT-BIH arrhythmia database (Moody and Mark, 2001). SLRF preprocesses the input signals by representing the input matrix as a combined sparse low-rank matrix and Gaussian noise matrix. This approach minimizes the squared difference between the denoised signal and the original signal. The nuclear norm captures the low-rank characteristics of the ECG signals, which preserves their overall structure and trends. The sparse regularization term also facilitates effective extraction of signal features, reducing noise interference. Module (b) involves feature extraction from ECG signals using MTF. We convert the one-dimensional time series signals into two-dimensional images to capture spatiotemporal features. First, we discretize the time series and map it to quantile intervals. Next, we construct a Markov transition matrix using a first-order Markov chain, and then expand it to MTF by incorporating spatial location information from the time series data. This process ultimately generates two-dimensional feature maps for model training, allowing for more comprehensive signal capture. Module (c) uses the BiFormer model to classify ECG signals. This model incorporates BRA. To reduce computational complexity, we divide the input two-dimensional feature maps into multiple non-overlapping regions, each with several feature vectors. We subsequently generate queries, keys, and values via linear projection, and then construct an affinity graph based on inter-regional relationships to determine each region’s relevance to the others. To filter out redundant information, the BiFormer model performs refined attention calculations on selected regions, with a focus on the most informative features. The detailed structure of the BiFormer block is shown on the right side of module (c). At the beginning of each BiFormer block, a 3 × 3 depthwise convolution is used to implicitly encode relative positional information, enabling the model to understand the relationships between differing locations. The model then sequentially harnesses the BRA module and the MLP module to achieve cross-location modeling and enhance each location’s feature representation capability, striking a balance between performance and computational complexity. The local context enhancement further optimizes the extracted features, improving the model’s ability to capture both local and global patterns. The BiFormer model employs a four-stage pyramid structure, where the spatial resolution of the input gradually decreases, while the number of feature channels increases with network depth. In the first stage, overlapping patch embedding is used to better capture local information. In the second through fourth stages, the network’s width (basic channel count, C) and depth are adjusted, and a patch merging module progressively reduces the spatial resolution of the input while increasing channel count. This design aims to reduce computational complexity while preserving key information. The optimized features are then fed into the classification layer for precise ECG signal classification. This entire process ensures the is capable of recognizing complex signals and achieves a balance between computational efficiency and model performance.

### 2.1 Dataset

To conduct a comparative study with existing classification models, we selected the widely used MIT-BIH arrhythmia database for ECG signal processing. This dataset includes 30 min of dual-channel ECG recordings from 48 patients, each recorded at a sampling rate of 360 Hz during a 30 minute run of Holter monitoring, providing approximately 110,000 annotated heartbeat instances. In this study, we performed data selection by excluding records 102, 104, 107, and 217, as they contain paced beats, which differ significantly in morphology and characteristics from normal heartbeats, making them unsuitable for the focus of our analysis. Each ECG sample includes two leads, one of which is MLII, while the second lead is one of the following: V1, V2, V4, or V5. For the purpose of this study, only the MLII single lead was utilized. To enable effective detection of PVCs, the MIT-BIH arrhythmia database was categorized into three classes: normal beats (N), PVCs (V), and other types. The classification scheme follows the AAMI standard and PhysioNet guidelines, as detailed in Table 1.

**TABLE 1:** Categories of the MIT-BIH arrhythmia database.

AAMI EC57 heart beat class	MIT-BIH heartbeat types	Number	Our classification
N	Normal beat (N)	74,546	N type
	Left bundle branch block (L)	8,075	Other types
	Right bundle branch block (R)	7,259	Other types
	Atrial escape beat (e)	16	Other types
	Nodal (junctional) escape beat (j)	229	Other types
S	Atrial premature beat (A)	2,546	Other types
	Aberrated atrial premature beat (a)	150	Other types
	Nodal (junctional) premature beat (J)	83	Other types
	Supraventricular premature beat (S)	2	Other types
V	Premature ventricular contraction (V)	6,903	V type
	Ventricular escape beat (E)	106	Other types
F	Fusion of ventricular and normal beat (F)	803	Other types
Q	Paced beat (P)	0	Other types
	Fusion of paced and normal beat (f)	0	Other types
	Unclassified beat (U)	15	Other types

We used a weighted loss function to handle the class imbalance problem. Specifically, we adjusted the weight parameter of the CrossEntropyLoss function using inverse frequency weighting, where the weights are inversely proportional to the frequency of each class. The weight calculation formula is as follows: ${\text{weight}}_{i}=\frac{N_{\text{total}}}{N_{i}}$ , where $N_{\text{total}}$ = 100,733 is the total number of samples across all classes, and *Ni* is the number of samples in class *i*. Using this method, the calculated weights are approximately, ${\;\text{weight}\;}_{N}$ ≈1.35, ${\text{weight}}_{V}$ ≈14.59, ${\text{weight}}_{\text{other}}$ ≈5.22. Finally, the weighted loss function adjustment is implemented as follows: “weights = torch.tensor ([1.35, 14.59 5.22]).float ().to (device); nn.CrossEntropyLoss (weight = weights); ”

To prevent model overfitting, we implemented a ten-fold cross-validation strategy (Awale and Reymond, 2018). Specifically, the dataset was randomly partitioned into ten subsets using stratified sampling to ensure that the class distribution within each subset was consistent with that of the entire dataset. In each fold of the 10-fold cross-validation, nine subsets were used for training and the remaining one for validation. The dataset comprises a total of 100,733 samples, resulting in approximately 10,073 samples in each test fold. Based on the dataset distribution, the N type accounts for approximately 74%, the V type accounts for about 6.8%, and the other types account for about 19.2%. Therefore, there are approximately 7,454 samples of the N type, 685 samples of the V type, and 1,934 samples of the other types in the dataset. The cross-validation procedure was repeated ten times, with each subset serving as the validation set once, ensuring robust model evaluation. Importantly, the training and validation sets were constructed from ECG recordings of different individuals to prevent any overlap of patient data between training and testing, thereby minimizing the risk of information leakage and overfitting. In each iteration, the model was trained on the training set and evaluated on the corresponding validation set. Upon completion of all iterations, the evaluation metrics from each fold were aggregated and averaged to obtain the final performance assessment of the model.

Experiments were conducted using a single GeForce RTX 4090 (24 GB) GPU within the framework of PyTorch 2.5.1, CUDA 12.4, and a CPU (16 vCPU Intel(R) Xeon(R) Platinum 8352V CPU @ 2.10 GHz). The hyperparameters used were: epoch = 100, batch size = 64, dropout = 0.2, depth = [2, 2, 8, 2], embed_dim = [64, 128, 256, 512], and mlp_ratios = [3, 3, 3, 3]. The specific model performance metrics are shown in Table 2.

**TABLE 2:** Model performance metrics.

Params	FLOPs	Throughput images/second	Training time (s)/Epoch
13.14 M	2.23 G	92.52	23.67

### 2.2 Pre-processing

Various factors, including baseline drift, electrode displacement, respiratory movements, and muscle tremors, can interfere with ECG data collection, distorting the ECG waveform and adversely affecting subsequent analyses (Satija et al., 2017; Kumar et al., 2021). Thus, denoising ECG signals is important prior to model classification and detection.

Here, we used SLRF (Karthikeyani et al., 2024) to denoise ECG signals. By combining sparse representation and low-rank decomposition, SLRF enhances ECG signal quality. The method’s sparse representation ensures that critical features within the ECG signals are preserved, while the low-rank decomposition captures varying trends within the ECG data. Together, these approaches provide a comprehensive method for denoising ECG signals.

For an input matrix $X\in R^{p\times q}$ , where R denotes the set of real numbers, the entry-wise squared norm ${∥X∥}_{e}^{2}$ and the entry-wise L1 norm $∥Y{∥}_{1}$ are defined as shown in Equation 1:

$${∥X∥}_{e}^{2}:=\sum_{i,j}\;{\left|X_{i,j}\right|}^{2}and{∥X∥}_{1}:=\sum_{i,j}\;\left|X_{i,j}\right|$$ (1)

where the nuclear norm is denoted as ${∥X∥}_{*}$ , and is represented by the singular values matrix X , as shown in Equation 2:

$${∥X∥}_{*}:=\sum_{i=1}^{h}\;\sigma_{i}\left(X\right)$$ (2)

where ${\;\sigma }_{i}\left(X\right)$ denotes the singular values of matrix $X\in R^{p\times q}$ , where h = min ( p,q ). The nuclear norm L1 relaxes both non-convex positioning and sparsity constraints, enabling greater flexibility in managing complex constraints during the optimization process. When the nuclear norm is applied to a singular value matrix, it is equivalent to the L1 -norm. This equivalence provides a powerful tool for low-rank matrix reconstruction and sparse representation, and enhances computational efficiency and stability when processing high-dimensional data.

A noisy matrix $X\in R^{p\times q}$ can be expressed as X=Y+W , where $Y\in R^{p\times q}$ represents a sparse, low-rank matrix, and $W\in R^{p\times q}$ represents a Gaussian noise matrix. To effectively estimate Y , the resulting optimization problem should be solved to promote both sparsity and low-rank characteristics, as detailed in Equation 3:

$${\mathrm{argmin}}_{Y\in R^{p\times q}}\left\{0.5∥X-Y{∥}_{e}^{2}+\lambda_{\circ }∥Y{∥}_{*}+\lambda_{1}∥Y{∥}_{1}\right\}$$ (3)

where $\lambda_{i}=\alpha_{i}\;\sigma ⩾0\left(i=0,1\right)$ represents the regularization factors and σ denotes the noise standard deviation. λᵢ is computed based on the noise standard deviation σ and the corresponding weighting coefficient αᵢ for the element-wise L1 norm $∥Y{∥}_{1}$ and the nuclear norm $∥Y{∥}_{*}$ as well as the corresponding weighting coefficient αᵢ, following the formulation: $\lambda_{i}=\alpha_{i}\sigma ⩾0$ . The noise standard deviation σ is automatically estimated from the input based on the actual noise level, while αᵢ is a trainable model parameter that is adaptively updated during the training process. The parameters $\alpha_{i}\left(i=0,1\right)$ are fine-tuned to maximize the signal-to-noise ratio within the sparse low-rank method.

### 2.3 ECG feature extraction

The Markov Transition Field (MTF) (Wang and Oates, 2015) is a method that transforms one-dimensional vibration signals into two-dimensional images using Markov transition probabilities. These two-dimensional images represent the complex structure and dependencies within the time series in a spatial manner, allowing for a more intuitive visualization of the signal’s periodicity, trends, correlations, and other features. This approach enables effective capture of ECG characteristics. It allows for more precise extraction of spatiotemporal features from local patterns and efficiently captures global patterns and long-range dependencies, thus enhancing the effectiveness of model training.

To effectively adapt time-series data to the Markov model, continuous data must be discretized. Each dimension of the time-series data $X=\left\{x_{1},x_{2},x_{i},\cdots x_{n}\right\}$ is divided into Q quantile bins. By identifying the quantiles, each value ​ $x_{i}$ is mapped to the corresponding quantile $q_{i}$ , and a Q×Q adjacency-weighted matrix (the Markov transition matrix) is constructed. The quantiles are then converted into the Markov transition matrix *W* using a first-order Markov chain along the time axis, as expressed in Equation 4:

$$W=\left[\begin{array}{ccc} w_{11} & \cdots & w_{1Q}\\ w_{21} & \cdots & w_{2Q}\\ \vdots & \ddots & \vdots \\ w_{Q1} & \cdots & w_{QQ} \end{array}\right]$$ (4)

where $w_{ij}$ represents the probability that quantile $q_{i\;}$ follows quantile $q_{j}$ , $w_{ij|P\left(x_{t}\in q_{i}|x_{t-1}\in q_{j}\right)}$ .

The Markov transition matrix assumes that state transitions depend only on the current state, ignoring the conditional relationship between the time series and the time step dependency (Abdel-Galil et al., 2004). In contrast, the MTF both considers the transition probabilities between states and incorporates spatial positional information. By arranging the transition probabilities in chronological order, MTF extends the concept of the Markov transition matrix, and constructs a more comprehensive Markov transition field *M*, as expressed in Equation 5:

$$M=\left[\begin{array}{ccc} m_{\left.ij\right|x_{1},x_{1}} & \ldots & m_{\left.ij\right|x_{1},x_{n}}\\ m_{\left.ij\right|x_{2},x_{1}} & \ldots & m_{\left.ij\right|x_{2},x_{n}}\\ \vdots & \ddots & \vdots \\ m_{\left.ij\right|x_{n},x_{1}} & \ldots & m_{\left.ij\right|x_{n},x_{n}} \end{array}\right]$$ (5)

where $m_{ij}$ denotes the transition probability from quantile $q_{i}$ to quantile $q_{j}$ , $m_{\left.ij\right|x_{i}\in q_{i},x_{j}\in q_{j}}$ .

### 2.4 ECG classification model

BiFormer (Zhu et al., 2023) achieves an improved balance between computational efficiency and performance by incorporating sparsity and adopting the BRA mechanism. The traditional ViT, due to its self-attention mechanism, experiences a significant increase in computational complexity, especially with large-scale inputs. As the image size and model parameters increase, the computational cost grows accordingly. This makes ViT computationally expensive and less efficient when handling high-resolution images or large-scale datasets. Particularly in the context of complex tasks, the computational burden becomes a bottleneck. In contrast, BiFormer significantly reduces computational complexity by introducing sparsity and the BRA mechanism. Specifically, BiFormer employs a query-adaptive sparse attention mechanism that selects the most relevant key-value pairs at a coarse-grained, regional level, avoiding the need to process key-value pairs at every position. This significantly reduces the computational load. By doing so, BiFormer improves computational efficiency while maintaining model performance. Especially when dealing with complex tasks, BiFormer makes more efficient use of computational resources and focuses on the most critical components, leading to improved performance. Overall, BiFormer achieves a better balance between computational efficiency and accuracy by reducing computations for irrelevant parts, resulting in optimized trade-offs between computational load and performance.

Through sparse attention and region-level processing, BRA reduces computational complexity and optimizes memory access. The expression for the total computation is shown in Equation 6:

$$\begin{array}{cc} & \;FLOPs={FLOPs}_{proj}+{FLOPs}_{routing\;}+{FLOPs}_{attn\;}\\ & \;=3HWC^{2}+2{\left(S^{2}\right)}^{2}C+2HWk\frac{HW}{S^{2}}C\\ & \;=3HWC^{2}+C\left(2S^{4}+\frac{k{\left(HW\right)}^{2}}{S^{2}}+\frac{k{\left(HW\right)}^{2}}{S^{2}}\right)\\ & \;\geq 3HWC^{2}+3C{\left(2S^{4}\cdot \frac{k{\left(HW\right)}^{2}}{S^{2}}\cdot \frac{k{\left(HW\right)}^{2}}{S^{2}}\right)}^{\frac{1}{3}}\\ & \;=3HWC^{2}+3Ck^{\frac{2}{3}}{\left(2HW\right)}^{\frac{4}{3}} \end{array}$$ (6)

where *H* represents the height of the input feature map, *W* represents the width of the input feature map, *C* is the token embedding dimension, and *k* is the number of involved regions. For this formula, the FLOPs are composed of three parts: *FLOPs*_*proj*_ (projection of Query, Key, and Value), *FLOPs*_*routing*_ (routing operation), and *FLOPs*_*attn*_ (token-to-token attention).

For *FLOPs*_*proj*_ *,*in the multi-head self-attention mechanism (MHSA), the Query, Key, and Value are derived through linear transformations from the input feature map (typically a tensor in the embedding space). The computational cost of these projection operations involves matrix multiplication, specifically:

The input feature map is a tensor of size *H* × *W* × *C*, where *H* and *W* represent the height and width of the image, respectively, and C denotes the embedding dimension. When computing MHSA, we need to map the input feature map to the spaces of Query, Key, and Value. The computational cost for each projection operation is H × W × C × C, as each input channel is mapped to a new embedding space.Since there are three projection operations—Query, Key, and Value—the total computational cost is given by Equation 7:

$$FLOPs_{proj}=3HWC^{2}$$ (7)

For *FLOPs*_*routing*_ *,*the routing operation is a distinctive feature of BiFormer, which leverages a regional similarity map to perform dynamic routing. In this process, the similarity between each pair of regions is computed, and the top-*k* most relevant regions are selected based on these similarity scores. Specifically:

Assuming the image is divided into *S* ^2^ regions, the computational complexity of calculating the similarity map between all regions is (*S* ^2^) × (*S* ^2^). Each region has a spatial size of *H* × *W*, and for each region, the computational cost is proportional to the embedding dimension *C*. The similarity map between regions involves all regions, and thus requires the use of *C*-dimensional features for computation. Specifically, two matrix multiplications are needed: one to compute the pairwise similarity between regions, and another to select the most relevant regions. Accordingly, the *FLOPs* required for the routing computation are summarized in Equation 8:

$$FLOPs_{routing}=2{\left(S^{2}\right)}^{2}C$$ (8)

For *FLOPs*_*attn.*_ *,* after the routing operation, each region selects *k* tokens, which are subsequently fed into the attention mechanism. Given that each region contains $\frac{HW}{S^{2}}$ spatial positions, self-attention computations are performed at each position within the region. Here, the attention computation involves *H* × *W* × *k* tokens (where *H* and *W* denote the height and width of the image, respectively), and the computational cost is proportional to the embedding dimension *C* of each token.

Additionally, since the size of each region is *S* ^2^, and each token will compute similarity with *k* tokens, the overall computational complexity is summarized in Equation 9:

$$FLOPs_{attn}=2HWk\frac{HW}{S^{2}}C$$ (9)

The factor of two arises because each attention operation requires computing the dot product between the query and key.

To reduce redundant computations, the workload is decreased through region partitioning, as expressed in Equation 10:

$$S={\left(\frac{k}{2}{\left(HW\right)}^{2}\right)}^{\frac{1}{6}}$$ (10)

where *S* represents the region partition factor. By adjusting the region partition factor based in the above formula, BRA achieves a computational complexity of $O\left({\left(HW\right)}^{\frac{4}{3}}\right)$ , which is lower than that of the original vanilla attention. For better understanding, a representative example of vanilla attention is the multi-head self-attention mechanism commonly used in the Transformer architecture (Vaswani et al., 2017), as shown in Equation 11:

$$\begin{array}{l} \text{Attention}\left(Q,K,V\right)=\text{softmax}\left(\frac{{\text{QK}}^{T}}{\sqrt{C}}\right)V,\\ \text{MHSA}\left(X\right)=\text{Concat}\left({\text{head}}_{0},{\text{head}}_{1},...,{\text{head}}_{h}\right)W^{o},\\ {\text{head}}_{i}=\text{Attention}\left({\text{XW}}_{i}^{q},{\text{XW}}_{i}^{k},{\text{XW}}_{i}^{v}\right) \end{array}$$ (11)

where Q, K, and V represent the query, key, and value matrices, respectively; softmax is used to assign weights to all keys for each query; a scalar factor $\sqrt{C}$ is introduced to avoid weight concentration and gradient vanishing; ${\text{head}}_{i}$ is the output of the *i*th attention head; $W_{i}^{q},W_{i}^{k},W_{i}^{v}$ are the corresponding input projection weights, and an additional linear transformation with weight matrix $W^{o}$ is used to combine the outputs of all attention heads, where the computational complexity is $O\left({\left(HW\right)}^{2}\right)$ , and BRA offers a more advantageous complexity in comparison.

To implement region partitioning and alleviate memory pressure, we first divided a two-dimensional input feature map *X* into *S × S* non-overlapping regions, such that each region contained $\frac{HW}{S^{2}}$ feature vectors. We then used a linear projection to derive the query, key, and value tensors *Q*, *K*, and *V*, as shown in Equation 12:

$$Q=X^{r}W^{q},K=X^{r}W^{k},V=X^{r}W^{v}$$ (12)

where $W^{q},W^{k},$ and $W^{v}$ are the projection weights for the query, key, and value, respectively. r refers to the “reshaped” version of the input matrix X.

Region-to-region routing with directed graphs determined the attention relationships between regions. By averaging the features within each region, we obtained the region-level query and key matrices $Q^{r}$ and $K^{r}$ . Then, by multiplying $Q^{r}$ with the transposed $K^{r}$ , we calculated the adjacency matrix of the region-level affinity graph, as shown in Equation 13:

$$A^{r}=Q^{r}{\left(K^{r}\right)}^{T}$$ (13)

where $A^{r}$ represents the degree of association between regions.

The entries in the adjacency matrix $A^{r}$ measure the semantic similarity between two regions. Next, the affinity graph is pruned, retaining only the top-k connections for each node, resulting in the routing index matrix, as shown in Equation 14:

$$I^{r}=topkIndex\left(A^{r}\right)$$ (14)

where $I^{r}$ represents the indices of the top *k* strongest connections between each region and the other regions.

Using the region-to-region routing index matrix $I^{r}$ , we can perform fine-grained token-wise attention operations. For each query token in region *i*, it will attend to the key-value pairs of the *k* routing regions indexed by $I^{r}$ (*i*,1), $I^{r}$ (*i*,2), ., $I^{r}$ (*i*,k). Since each query accesses all the key-value pairs of the routing regions, in order to improve memory access efficiency, we can pre-collect the key-value pair tensor, as shown in Equation 15:

$$K^{g}=gather\left(K,I^{r}\right),V^{g}=gather\left(V,I^{r}\right)$$ (15)

where $K^{g}$ and $V^{g}$ are the key and value tensors.

To make the model more efficient when handling high-resolution or large-scale data, we applied fine-grained token-to-token attention to the collected key-value pairs, as shown in Equation 16:

$$O=Attention\left(Q,K^{g},V^{g}\right)+LCE\left(V\right)$$ (16)

where *LCE* refers to the local context enhancement term.

After performing the attention operation, the output features were fed into the classification layer, which extracted and used key features from the ECG signal to achieve accurate data classification.

## 3 Results

### 3.1 Eigenvalue analysis

**FIGURE 2:**
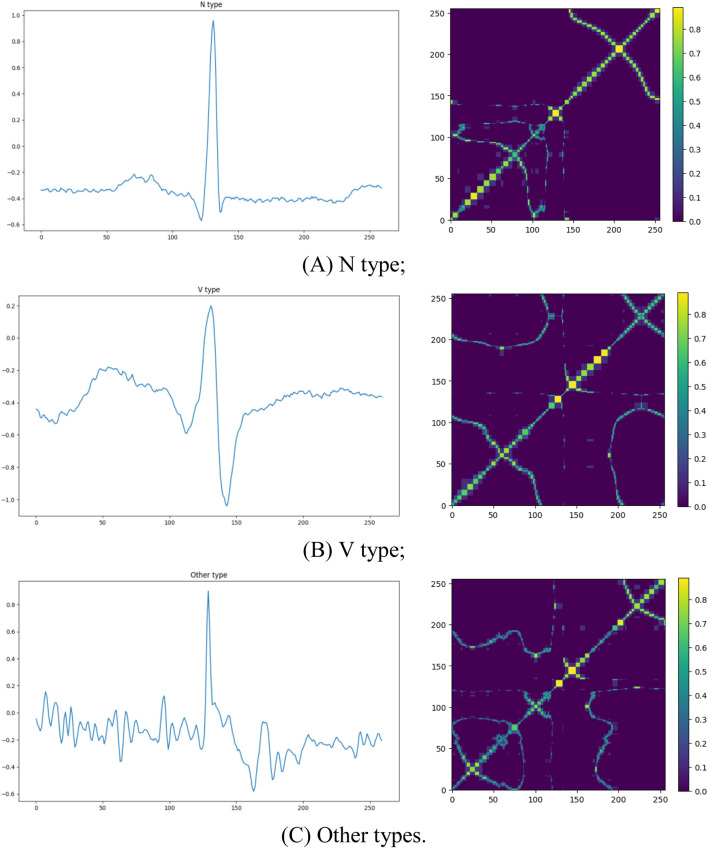
The original ECG signal plots and corresponding MTF images for different categories. The left side shows the raw ECG signals, and the right side displays the corresponding MTF representations: **(A)** N type; **(B)** V type; **(C)** Other types.

Here, we visualized the original signals and their corresponding MTF representations for different categories of ECG signals, as shown in Figure 2. The left panel displays the raw ECG signals, while the right panel presents the corresponding MTF images. The direction of signal transitions reflects the trend of changes in the ECG signal. In the normal ECG category (N), electrical cardiac activity follows a regular sequence, and the transition flow is orderly and continuous, indicating normal cardiac function. In contrast, in the two abnormal ECG categories, the transition flow is disordered and/or chaotic, with loops, reverse flows, delays, or jumps, all reflecting abnormal cardiac electrical activity. This result suggests that MTF images can capture significant differences between ECG signal types, providing strong visual evidence for abnormal ECG detection and laying the foundation for subsequent classification tasks.

### 3.2 Performance evaluation

We compared relevant studies based on the MIT-BIH arrhythmia database and evaluated the performance of BiFormer against seven recently proposed algorithms. In this study, we adopted micro-average as the primary performance evaluation metric. The class distribution in the MIT-BIH arrhythmia database is imbalanced, with normal beats significantly outnumbering abnormal types such as PVC. Micro-average calculates the prediction results across all classes uniformly, which better reflects the model’s overall performance on the entire dataset and provides a more comprehensive assessment of the detection system’s overall classification ability. Accuracy, specificity, recall, and F1 score are commonly used evaluation metrics for classification models. Accuracy represents the proportion of correctly predicted samples to the total number of samples, as shown in Formula 17:

$$\text{Accuracy}=\frac{TP+TN}{TP+TN+FP+FN}$$ (17)

Specificity measures the proportion of actual negative samples that are correctly predicted as negative, as shown in Formula 18:

$$\text{Specificity}=\frac{TN}{TN+FP}$$ (18)

Recall measures the proportion of actual positive samples that are correctly predicted as positive, as shown in Formula 19:

$$\text{Micro}\_R=\frac{\sum_{i=1}^{n}{\text{TP}}_{i}}{\sum_{i=1}^{a}\text{TP}+\sum_{i=1}^{a}{\text{FN}}_{i}}$$ (19)

The F1 score is the harmonic mean of precision and recall, providing a balanced consideration of both metrics, as shown in Formula 20:

$$\text{Micro}\_F1=\frac{2\times \frac{\sum_{i=1}^{n}TP_{i}}{\sum_{i=1}^{n}{\text{TP}}_{i}+\sum_{i=1}^{n}\text{FP}}\times \text{Micro}\_R}{\frac{\sum_{i=1}^{n}TP_{i}}{\sum_{i=1}^{n}{\text{TP}}_{i}+\sum_{i=1}^{n}\text{FP}}+\text{Micro}\_R}$$ (20)

The experimental results are shown in Table 3. BiFormer achieved the best performance across multiple metrics, with an accuracy of 99.45%, specificity of 99.81%, recall of 99.89%, and an F1-score of 98.86%. Compared to existing methods, BiFormer showed a 0.33% improvement in accuracy. While other algorithms performed well in ECG classification, our model exceled in capturing both global and local information, significantly improving the effectiveness of signal classification. This finding highlights BiFormer’s remarkable adaptability and potential.

**TABLE 3:** Performance comparison table between our model and existing models.

Methodology	Accuracy	Specificity	Recall	F1-score
MS-DSwin-AL (Karthikeyani et al., 2024)	95.72	94.81	93.64	89.53
RBFNN (Kishore et al., 2022)	97.85	98.15	96.61	96.84
CNN + LSTM (Madan et al., 2022)	97.98	97.40	97.84	98.39
XAI (Raza et al., 2022)	98.25	97.87	98.44	97.42
CWGAN-GP (Ma et al., 2022)	98.68	98.72	97.39	97.06
MPA-CNN (Houssein et al., 2021)	98.95	96.84	98.13	98.37
HARDC (Islam et al., 2023)	99.12	98.61	98.73	98.51
Proposed work	99.45	99.81	99.89	98.86

**FIGURE 3:**
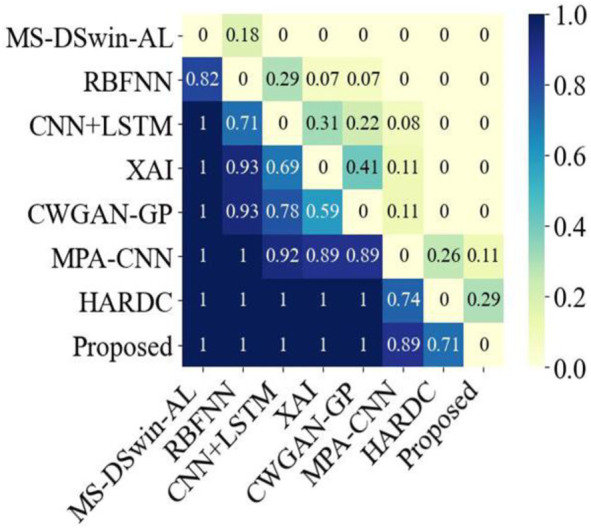
Bayesian analysis comparison between the proposed method and the seven latest current methods.

Bayesian analysis (Lin et al., 2010) is a probabilistic evaluation method that is used to quantitatively compare performance differences between algorithms. It first assumes that the performance differences between any two algorithms follow a normal distribution, and then selects a prior distribution to express initial beliefs about these differences. Then, using actual data, the probability of observing the current data for a range of difference values is calculated. The posterior distribution is obtained via Bayes’ theorem, which intuitively quantifies the performance differences between the two algorithms. We used Bayesian analysis to evaluate the performance differences between BiFormer and other algorithms (Figure 3). These results further demonstrated that our novel method had exceptional performance in multiple comparative experiments, outperforming the latest algorithms in the vast majority of cases.

**FIGURE 4:**
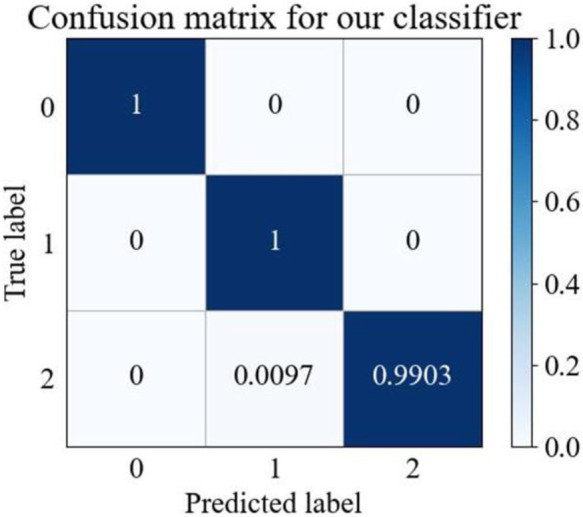
MTF-BiFormer confusion matrix. The X and Y-axes: 0 represents N type, one represents V type, and two represents Other types.

Confusion matrices (Valero-Carreras et al., 2023) can be used to evaluate classification model performance, as they illustrate the comparison between predicted and actual results for different categories, and reveal a given model’s performance across these categories. As shown in Figure 4, our proposed model effectively differentiated between “N type” and “V type” categories, with all samples accurately predicted. Additionally, the model demonstrated exceptional accuracy in identifying the “Other types” category, with 99.03% of samples correctly classified. These results indicate that the proposed method shows stability and superior performance in ECG classification.

### 3.3 Feature visualization

**FIGURE 5:**
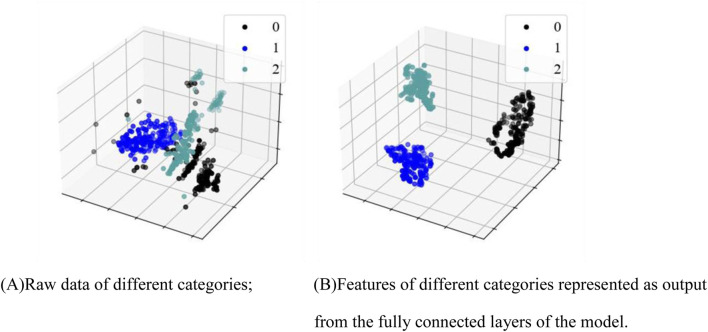
Feature visualization map. **(A)** Raw data of different categories; **(B)** Features of different categories as output from the fully connected layers of the model.

The t-distributed Stochastic Neighbor Embedding (t-SNE) (Wang et al., 2018) is a commonly-used dimensionality reduction technique that transforms the similarities of high-dimensional data into probability distributions, mapping the data into a lower-dimensional space for feature visualization. The t-SNE visualization of ECG data before and after classification is shown in Figure 5, where 0 represents the N type, 1 represents the V type, and 2 represents Other types. As can be seen in Figure 5A, the three types of ECG signals exhibited scattered and disordered distributions, indicating that their similarities were not prominent in the high-dimensional space. In contrast, as Figure 5B shows, there was clearly a separation between the three types of signals in the lower-dimensional space.

Clinically, normal ECG, PVC ECG, and other types of ECG represent different types of electrocardiographic signals, and the differences between them are naturally reflected in the electrical activity patterns of the signals. Normal ECG reflects the regular electrical activity of the heart, with stable waveform morphology. The characteristics of normal ECG signals lead to relatively consistent features across samples. In contrast, PVC and other types of ECG differ from normal ECG. t-SNE, by comparing and distinguishing these signal features, is capable of identifying the different characteristics of these signals and grouping them into relatively independent clusters. On the algorithmic level, t-SNE is an unsupervised learning method, particularly adept at reducing the dimensionality of data while preserving its local structure. By calculating the similarity between data points, it groups similar signals together in a low-dimensional space, while separating signals from different categories. The core idea of t-SNE is to maintain the local structure of the data, preserving the relative distances between data points as much as possible. When signals exhibit similarity in certain features, t-SNE maps these signals to adjacent positions in the low-dimensional space. Consequently, the similarity of normal ECG signals (the regularity of their waveforms) causes them to cluster together in the low-dimensional space, while PVC and other types of ECG signals, having distinct characteristics, are separately clustered by t-SNE. These findings suggest that our algorithm effectively captured the feature differences between different categories of ECG signals. They also confirm that the features of classified ECG signals are distinguishable, supporting the effectiveness of our proposed PVC detection method.

### 3.4 Ablation study

We conducted an ablation study to evaluate the performance of different variants on the ECG classification task. As shown in Table 4, the results demonstrate the superior performance of the combination of MTF and BiFormer. MTF, by revealing the state transitions, temporal dependencies, and dynamic changes of the signal, effectively models the dependencies within the time series. The spectrogram reflects the periodicity, variability, and high-frequency components of the ECG signal, and its performance is second only to MTF. In contrast, the wavelet transform captures the signal’s variation across different frequency scales but contains less information. While the raw ECG signal image includes comprehensive data, much of the key information is actually embedded within the complex patterns and noise of the signal, making feature extraction directly from the raw signal more challenging and impacting classification performance. Furthermore, when retaining the MTF method, we replaced BiFormer with the Swin transformer for comparison, and the proposed method still achieved superior performance.

**TABLE 4:** Ablation study of different variants on the ECG classification task.

Method	Accuracy	Specificity	Recall	F1
Raw ECG images	86.39%	81.95%	91.26%	88.55%
Wavelet scalograms	95.19%	98.64%	97.85%	96.92%
Spectrogram	96.71%	98.25%	95.38%	97.54%
MTF + Swin transformer	98.18%	98.05%	97.94%	98.26%
Proposed work	99.45%	99.81%	99.89%	98.86%

## 4 Discussion

### 4.1 Comparison with other feature extraction

Feature extraction is a critical component of signal processing which aims to extract useful information from signals. Over the past several years, an increasing number of researchers have chosen to convert one-dimensional ECG signals into two-dimensional images prior to classification, allowing for a more comprehensive capture of signal features. ECG signals primarily reflect cardiac activity via a variety of components, including P waves, QRS complexes, and T waves, all of which are represented by temporal variations. Therefore, capturing the temporal features of these signals is crucial for effective ECG feature extraction. Yang et al. (2021), effectively extracted deep features related to lead correlation and independence in ECG signals from the time domain. They proposed a dual-channel hybrid convolutional neural network for feature extraction called THC-Net, which incorporates two structures: the Canonical Correlation Analysis (CCA)-Principal Component Analysis (PCA) convolutional network and the Independent Component Analysis (ICA)-PCA convolutional network. The former maximizes the correlations between leads through linear combinations in order to capture the relationships between different leads, while the latter decomposes the each lead’s signals into a set of independent components to capture independent lead features. The combination of these two structures allows for a more comprehensive ECG signal capture. There have also been been numerous experiments which have combined time and frequency domains for ECG signal feature extraction. Wang et al. (2022), combined time and frequency domains for ECG feature extraction by first using R-wave localization to segment each heartbeat cycle. They then applied Fast Fourier Transform (FFT) to extract frequency domain information for each heartbeat cycle, and by concatenating the extracted time-domain and frequency-domain information, captured and analyzed the signal. Although decomposing the signal into different frequency components allows for feature extraction from multiple angles, frequency domain analysis typically requires a longer signal window, which may result in information loss related to the original time domain, as well as higher computational complexity. During PVCs, ECG signal wavelengths change rapidly, but time-domain methods can capture these rapid changes, improving PVC identification accuracy.

MTF is a popular image-encoding method that treats time sequences as Markov processes. Based on the time domains, the Markov processes are divided into multiple quantile bins to construct a Markov transition matrix, capturing the transition probabilities and dynamic correlations between different signal bands and enabling a more comprehensive extraction of ECG signal features in the time domain. By capturing the transitional patterns between different states throughout the time series, MTF also indirectly reflects signal frequency changes, where high-frequency signals typically lead to more frequent state transitions, while low-frequency signals result in fewer transitions. Additionally, MTF considers the spatial variations of ECG signals, thus reflecting the state correlations between time points and describing the transition patterns of these states across different locations. By combining both time and spatial information, MTF can capture local details and global signal trends, providing more accurate information for ECG classification.

MTF, by modeling the temporal transition patterns of ECG signals and the dynamic relationships between states, is capable of capturing long-range dependencies and frequency variations in the signals, providing strong support for time-domain feature extraction. However, MTF primarily focuses on capturing global patterns and temporal dependencies of the signal, which, while effective for handling long-term dependencies, has limited ability to capture local details and abrupt changes within the signal. The introduction of BiFormer addresses this limitation. By combining MTF and BiFormer, the accuracy and robustness of ECG signal classification can be effectively enhanced.

### 4.2 Comparison with other classification models

Over the past several years, an increasing number of researchers have turned to classification models for ECG signal detection (Table 5). For example, Jahmunah et al. (2022) were able to successfully identify myocardial infarctions using the DenseNet model. This model employs a dense connection structure, where each layer is connected to all previous layers in the network rather than just the directly preceding layer, which improves classification efficiency and performance. Additionally, by applying enhanced class activation mapping (Grad-CAM) techniques, the model visualizes the ECG leads and waveform segments making classification decisions. However, the dense connection structure of DenseNet also leads to longer training times. Peng et al. (2022) combined three classification models to achieve effective arrhythmia classification. Their method used a one-dimensional convolutional neural network (CNN) to extract spatial features, and employed a bidirectional long short-term memory (Bi-LSTM) network to capture temporal features. It also harnessed a sequence-to-sequence (Seq2Seq) framework to handle input and output sequences of varying lengths. The introduced attention mechanism allowed the decoder to focus on the input parts that were most relevant to the output, which improved classification accuracy and model performance while also effectively addressing the complexity of spatiotemporal features and class imbalance in ECG data. However, the complexity of this model leads to relatively high computational costs. Hao et al. (2023), achieved good results in signal classification using an improved G2-ResNeXt model. To better capture the low-frequency characteristics of ECG signals, this model replaces the smaller convolution kernels in the original ResNeXt with larger ones, allowing for more comprehensive signal capture and the reduction of noise interference. It also uses hierarchical convolutions to progressively extract local and global features, which enhances classification capabilities. However, although G2-ResNeXt effectively extracts local spatial features, it may have insufficient robustness to noise. Qin et al. (2024) improved ECG signal classification performance using a Self-Organizing Neural Network (SelfONN). This model allows node operators to dynamically adapt and optimize depending on the specific connection weights that are generated during the training process, which improves generalizability when handling multi-label classification tasks. However, because the SelfONN network has a relatively fixed structure, it may be vulnerable to information loss or model overfitting when processing highly variable and complex data. Ge et al. (2024) employed a Graph Convolutional Network (GCN) in their classification model, and also used an ECG Knowledge Graph (ECG-KG) framework. By performing convolution operations on the graph structure of the knowledge graph, the model aggregates the feature information of neighboring nodes layer-by-layer, which effectively captures the complex relationships between different ECG waveform features. However, the model’s performance largely depends on the construction of the input graph structure. Although existing ECG signal classification methods have achieved certain results in their respective fields, they generally face some common challenges and limitations. Many methods, such as DenseNet and Bi-LSTM, involve complex network architectures, which lead to high computational costs and prolonged training times when processing large-scale ECG data. Additionally, while these methods can effectively extract spatiotemporal features, their ability to capture long-range dependencies is relatively weak. This is particularly problematic when dealing with complex cardiac diseases, where capturing sustained signal changes and effectively identifying early symptoms remains a significant challenge. Furthermore, ECG-KG, a framework based on graph convolutional networks, is capable of capturing complex relationships between waveforms; however, it is heavily dependent on graph structures, and its ability to handle high-dimensional data still faces considerable challenges.

**TABLE 5:** Comparison of advantages and disadvantages of different models.Performance.

Method	Advantage	Disadvantage
DenseNet (Jahmunah et al., 2022)	Enhance feature reuse to improve the model’s representation capability and learning efficiency	Long training time
CNN + Bi-LSTM + Seq2Seq (Peng et al., 2022)	Comprehensively extract spatial and temporal features of ECG signals to enhance classification accuracy and robustness	Low computational efficiency
G2-ResNeXt (Hao et al., 2023)	Effectively extract local features	Insufficient robustness to noise
SelfONN (Qin et al., 2024)	Strong generalization ability	High risk of overfitting
GCN (Ge et al., 2024)	It effectively captures local topological information in graph-structured data, particularly excelling at modeling the relationships between nodes and their neighbors	Strong dependency on graph structure

The Transformer architecture, by introducing the self-attention mechanism, has demonstrated significant advantages in addressing the aforementioned challenges. In vanilla attention, the MHSA is employed to compute the relationships between various elements in the input sequence. This process involves pairing each position in the input sequence with all other positions to generate corresponding attention weights, thereby overcoming the issue of long-range dependencies. Furthermore, compared to more complex models, the Transformer improves training efficiency through parallel computation, reducing computational costs and training time. However, the computational complexity of this method grows quadratically with the increase in input size, leading to significant scalability issues. To address this, our proposed BiFormer adopts a content-aware approach, computing only the key parts of the input sequence that are relevant to the current task. In each layer, BiFormer adaptively selects the paths that require deeper processing based on the input features, thereby reducing computational memory and enhancing the model’s performance. This provides a more comprehensive and innovative solution for ECG signal classification tasks.

### 4.3 Limitations and future research lines

In the field of signal analysis and detection, deep learning models typically rely on large amounts of labeled data to improve generalizability. However, ECG data collection is complex and time-consuming, as it requires ensuring the stability of ECG devices and active patient cooperation. Furthermore, due to factors such as age, gender, lifestyle, and health conditions, different patients’ ECG signals can exhibit significant variation. These individual differences can make it challenging for models to accurately recognize ECG features across diverse groups of patients, which affects their reliability and effectiveness when clinically applied.

In future research, we aim to establish a diverse ECG dataset and develop advanced data augmentation techniques to ensure that our model can effectively detect a wide range of ECG features. This will help us address the substantial individual differences that are present across different patient groups. We also plan to customize model training based on specific patient characteristics, and to explore the potential of personalized medicine in ECG analysis. By applying artificial intelligence to advance cardiologic research, we hope to contribute to further developments in medical technology and machine learning.

## 5 Conclusion

PVCs are an early warning sign of many serious heart conditions. Early and accurate detection of PVCs can help identify potential cardiac abnormalities, enabling timely intervention to prevent disease progression. Here, we combined MTF with a BiFormer classification model to achieve effective and rapid PVC detection. This approach aims to provide fast and efficient PVC detection, offering significant value for clinical applications.

We selected a portion of the MIT-BIH arrhythmia database as experimental samples. First, we applied SLRF to preprocess ECG signals, removing noise while preserving key waveform details. This method combines sparsity and low-rank techniques, making the filter more robust to handle various noise sources. Next, we used MTF for feature extraction. This method takes the transition probabilities between states into account and integrates spatial location information, thus effectively capturing spatiotemporal features. Our combination strategy improved the model’s training performance and significantly enhanced predictive accuracy, making the model more efficient, particularly for complex datasets. Finally, we used the BiFormer model for ECG signal classification. This model leverages dynamic adjustment of BRA mechanisms and adaptive sparse attention to accurately filter information. Additionally, with its region-based partitioning strategy, BiFormer maintains exceptional performance when handling high-resolution data. Our approach effectively balanced computational efficiency and model performance and achieved an overall accuracy of 99.45%. By applying the MTF, one-dimensional temporal ECG signals are transformed into two-dimensional images that preserve temporal dependency structures, effectively enhancing the representation of dynamic evolutions and key pathological features. Based on this, we adopt BiFormer as the backbone network, which leverages the BRA mechanism to conduct hierarchical feature modeling at both the regional and token levels. This design substantially improves the model’s sensitivity to both local waveform details and global rhythm characteristics, while also optimizing computational efficiency. The structured input provided by MTF is highly compatible with the sparse attention architecture of BiFormer, achieving deep synergy between feature representation and model structure. As a result, the proposed method enables accurate detection of PVCs in complex ECG signals, offering a promising approach for intelligent clinical diagnosis of PVCs.

## Data Availability

The original contributions presented in the study are included in the article/supplementary material, further inquiries can be directed to the corresponding authors.
